# A cross-sectional study on the physical and mental health condition of university newcomers in Bangladesh

**DOI:** 10.1007/s44192-025-00276-5

**Published:** 2025-08-20

**Authors:** Arpita Roy, Sadia Amrin, Md. Ashraful Alam, Hafsa Akter, Sadhon Kumar Ray, Md. Momin Islam, Munawar Sultana

**Affiliations:** 1 Department of Microbiology, University of Dhaka, Dhaka, 1000 Bangladesh; 2 Department of Meteorology, University of Dhaka, Dhaka, 1000 Bangladesh

**Keywords:** University students, Newcomers, Physical and mental health, COVID, Medicine, Bangladesh

## Abstract

In Bangladesh, an extensive enrollment examination is required to enroll in different universities, where students from all 64 districts of Bangladesh compete to get a place. Students either migrate from their home districts to another place or continue from home if they reside in the same city. However, it is undeniable that there is a huge and sudden change in the lifestyle of the newly enrolled students. This study was conducted on 50 universities in Bangladesh to investigate the overall health conditions of the university newcomers (N = 605) in six academic sessions from 2016 to 2022. The study showed that more than half of the students (52.6%) became physically sick after admission. The majority (81.5%) of the respondents felt mental pressure, and the maximum (83.3%) were concerned about their mental health condition, which is often unnoticed for this group of students. This also highlighted that during COVID-19, participants’ mental health deteriorated drastically (71.7%). Nevertheless, the medical centers of the respective institutions were completely heedless in the majority (79.3%) of cases. Lastly, the respondents themselves tried to provide appropriate suggestions that will be helpful for the future well-being management of the newcomers. Thus, unveiling the overall health condition of newcomers is necessary so that every respective individual becomes aware of the condition and the current situation changes into a student-friendly and lively environment. To our knowledge, this is one of the first kinds of investigation in Bangladesh.

**Supplementary Information:**

The online version contains supplementary material available at 10.1007/s44192-025-00276-5.

## Introduction

The term “Health” means the overall condition of the human body. The World Health Organization (WHO) defined health as “the state of complete physical, mental and social well-being and not merely the absence of disease and infirmity” in 1948. Again, in 1986, the definition was clarified by WHO as “A resource for everyday life, not the objective of living. Health is a positive concept emphasizing social and personal resources, as well as physical capacities.” This indicates that health is the arrangement that supports a person's role in the wider society rather than an end in itself. Health is also the ability of a body to adapt to new threats and infirmities [1]. The markers that are directly related to health conditions are physical and mental well-being; freedom from disease, pain, or defect; normalcy of physical and mental functions; and soundness.

Physical health indicates the normal functioning of the body. Physical health is a dynamic state characterized by the development of biochemical, physiological, and mental processes that influence labor capability and human social activities[2]. Physical health also defines the ability to take care of one's personal needs and the ability to work. Physical health has also a connection with the normal functioning of the body, enabling one to survive and avoid illness[2]. Sleeping and eating properly, physical activeness, maintaining proper hygiene, and getting enough relaxation are the aspects that are involved with physical health.[3]. Cardiovascular endurance, muscular strength, muscular endurance, flexibility, and body composition are the five elements of physical fitness[4].

On the other hand, mental health is associated with emotional, psychological, and social well-being. It directly affects how we think, feel, and act. It also determines how we handle stress, relate to others, and make health choices[5]. Mental health is important at every stage of life, from childhood and adolescence to adulthood[6]. The three major components of mental health include Cognitive Health, Emotional Health, and Behavioral Health. Each of these components affects each other and interacts with the overall well-being.

There are various factors affecting physical health conditions. Such as environment, climate, temperature, social culture, and place of living [7]. The environment can affect a person’s physical health. The surroundings have a drastic effect on a person’s health. Different elements of the environment together determine one’s health state. It affects one’s overall physiological processes. A healthy mind depends on physical well-being. A sound mind resides in a healthy body. The overall climate differs from one geographical location to another. Environmental factors play crucial role in the health condition of people. In a previous study, it was seen that students were more vulnerable to depression and anxiety who were not satisfied with the university culture. Besides, variations in the sleep cycle the amount of meal intake were also the reasons behind depression and anxiety, according to the study [8]. Climate change is another concerning factor that affects the health condition of people, like increasing morbidity and mortality rate [9] The climate of the humid region is different from the cold-bound region. A person living in a humid region cannot easily survive in a colder region. The human body takes a certain time to adapt to different climates[10]. Temperature can also affect a person’s physical and mental well-being. Our body has a specific temperature within which we can enjoy soundness. Below and above the temperature range can imbalance a proper body balance. The place of living affects a person’s health. A hygienic place encourages physical soundness and immunodeficiency. This makes a person’s attitude positive or negative. A person residing in unhygienic places often suffers from various diseases [11]. Socio-economic conditions are one of the leading factors affecting the physical and mental health of university newcomers. These factors directly or indirectly influence the academic performance of the students. In a previous study conducted on the public and national university students of Bangladesh, it was shown that monthly educational expenditure, the living place, library facilities, daily study- duration, extra-curricular activities, results of the exams and future planning after completing education were the determinants of socio-economic condition of the students [12]. The same study reported that most of the students' parents were farmers, as Bangladesh is an agricultural country. Another significant factor is monthly expenditure, which affects mental health conditions. Fathers' occupation and family status/structure are two of the most important factors that significantly affect the academic performance of students [13]. The present research differs from the previous ones with respect to the fact that in this research socio-economic conditions are not the sole focus but in depth analysis of physical and mental health conditions in context of variable factors of the newcomers during the fresher’s period has been emphasized.

Mental health and physical health are interconnected. The interrelation between these two is often neglected. A number of people are not conscious about mental disorders, let alone their treatment. Thus, physical problems strike at the highest form, and severe chronic life-threatening situations are observed. Regardless of age, poor health condition is directly or indirectly dependent on mental health condition. [22] Physical health sometimes affects one’s mental soundness. One may be more susceptible to mental health issues due to personal, psychological, and biological characteristics like emotional intelligence, substance use, and heredity. A person is more likely to develop mental health issues when they are exposed to adverse social, economic, geopolitical, and environmental conditions, such as poverty, violence, inequality, and environmental squalor. The state of one’s physical and mental health might get worse together. Physical health problems can start to develop as a result of mental health issues [14]. For instance, diabetes and other physical health issues might develop as a result of depression. On the other hand, a person who has a painful bodily ailment is more prone to eventually develop a mental health condition. Physical sickness may be more challenging to manage. The symptoms could get worse if these cycles continue.

This study is conducted on the overall physical and mental health condition of the university newcomers. There were previous studies on either the physical or mental health condition of universities [15]. However, no previous study was found that emphasized both the physical and mental health of university newcomers, especially in Bangladesh. Besides, available studies focused on total university students, not exclusively the newcomers who should be the priority group to manage long-term physical and mental trauma. Moreover, studies on the mental health condition of university students during the COVID-19 pandemic were considered here [16]. In the context of Bangladesh, this study will play a significant role. The physical and mental health of university students is the main target of this research. In the perspective of Bangladesh, the transition period of study is the tertiary level education known as the University's first year. This is called so because before entering university, students are under the full guidance of their parents and authority. But in tertiary education, the transition includes adjusting to different learning environments and assessment systems and teaching practices. Another study in Australian Universities focused on the transition based on social networking, social skills, and communication competence [17]. The above studies showed the transition at the University level based on different aspects, but none of them focused on the transition from secondary to tertiary level in Bangladesh. Moreover, the linguistic transition at the tertiary level in Bangladesh was mentioned in research [18]. This research will act as a baseline if any further studies related to this topic are done. A connection between good health and academics is important, and that too has been shown here. All of the abovementioned reasons justify the necessity of the study.

This study combined all of the above-mentioned cases for the first time as a survey report that projected key factors for physical illness of a huge portion of the participants, along with the impact of the COVID-19 pandemic on the overall health condition of students after being enrolled in university.

Here, we aimed to find out the possible reasons why and how the newcomers get affected physically and mentally, if there is any possible relation between their lifestyle and sickness if any particular reason is influencing their overall health condition, find possible solutions, and come up with suggestions given by the participants themselves in this study. This study is a very preliminary one in the aspect of Bangladesh, as we could not connect with all of the students of all the universities. Moreover, we also could not focus on the socioeconomic status of all the interviewees, as our main focus was not on this. However, we have planned to conduct this study overall in the country afterward.

## Methodology

This is a cross-sectional study mainly focused on the overall health status of university newcomers, and this involved a single occurrence of data collection with no follow-up. The study was performed from November 2022 to April 2023 using both an online-based self-administered way (Google form) and a printed version of the online form. Participating university students’ physical and mental health was evaluated based on the questionnaires provided in the form. The data-gathering process was designed to ensure quick responses. The questionnaire was administered to the respondents. The online questionnaire was sent to the students of the different universities using social media platforms such as Facebook and Messenger. A convenience sampling method was performed because it was accessible, quick and cost-effective to gather information. Moreover, time and access to the full population were limited. After all the data collection, data analysis was done. A Bivariate Chi-square test with significant *P* values was added. For analysis, Stata 17 was used as a tool.

### Questionnaire summary

In total, there were 60 questions in the questionnaire. It was divided into 4 sections. In the first section, the introduction of the participants was taken. Participants’ names, ages, genders, contact information, home districts, current academic sessions, and residences were included. The second portion emphasized the whole physical condition of the respondents. The number of times of the sickness, previous disease history, consultation, medication, food they intake, living place conditions, environmental factors, COVID-19 vaccination, and whether the respondents were affected by COVID-19 or not were the highlighted ones. The third portion was designed to focus on participants’ mental health. If they are aware of the importance of mental health, whether they face any problems related to this, their relationship with their parents, communication skills, and notable changes in their lifestyle after getting admission were the main concerns here. The last portion was for the respondents’ suggestions for the upcoming newcomers and consent whether they provided the data willingly or not. The majority of the questions in the questionnaire had multiple-choice options. In some questions, respondents were needed to write their information. The questionnaire was shared among the interviewees in both online and printed copies. Among 605 responses, 150 were collected from the printed copies. The rest of the 455 responses were gathered from an online Google form. The ratio was inconsistent because it was hard to reach different districts and institutions in person.

### Participants

The participants' selection process was random. No systematic sampling methods were used here.

#### Institutions

A total number of 605 students of Bangladesh from 50 different institutions (Table 1) participated in this survey where there were 28 different Universities, 11 different Medical Colleges, 6 Engineering Universities, and 5 Science and Technology Universities. Among 605 students, 523 participants (86.4%) were from universities. The number of participants from different Engineering Universities was 37 (6.1%). A total of 27 (4.5%) participants were from different Medical Colleges. Lastly, 18 (2.9%) students from Science and Technology Universities have participated.

**Table 1:** Demographic characteristics of the interviewee (N = 605)

Demographic parameters	No of participants (percentages)
*Gender*	
Male Female	293 (48.4%) 312 (51.6%)
*Age (years)*	
18–20 21–23 24–26	252 (41.7%) 345 (57.0%) 8 (1.3%)
*Institutions*	
Total no. of institutions Universities Medical Colleges Engineering Universities Science and Technology Universities	50 28–523 Students (86.4%) 11–27 Students (4.5%) 06–37 Students (6.1%) 05–18 Students (2.9%)
*Session*	
2021–22 2020–21 2019–20 2018–19 2017–18 2016–17	285 (47.1%) 220 (36.4%) 83 (13.7%) 06 (2.9%) 09 (1.4%) 02 (0.3%)
*Home district*	
Total no. of districts Dhaka Outside Dhaka	61 01–65 Students (10.8%) 60–540 Students (89.3%)
*Residence*	
Resident of Hall Non–resident of Hall Self-accommodated	235 (38.8%) 325 (53.7%) 45 (7.4%)

#### Academic Sessions

In total, 6 different academic sessions were covered (Table 1). As the purpose of this study was to focus on the newcomers, the majority of the people were from freshman year, and their session was 2021–22. The number was 285 (47.1%), and they shared their concerns. The number of participants from the 2020–21 session was 220 (36.4%). From the 2019 -20 session, 83 students (13.7%). 6 (2.9%) participants were from session 2018–19. 9 (1.4%) participants were from the session 2017–18. The least number of the population were from 2016–17, and only 2 participants (0.3%) were from this academic session. All of them were on the same tertiary level.

#### Gender

Both male and female respondents were part of this survey. The majority portion was female 312 (51.6%). The number of male participants was 293 (48.4%).

#### Age (years)

Altogether, 3 age ranges had been selected for conducting the study. Individuals were from 18 to 26 years old. 252 (41.7%) respondents were in the 18–20 age range. The majority were from the 21–23 years age range, 345 (57.0%) of the total population. From the age range of 24–26 years, only 8 (1.3%) responded.

#### Home districts

Among 64 districts, this survey could reach 61 districts. Inside the capital, Dhaka city, the total number of participants was 65 (10.8%). Outside Dhaka, the total number of respondents was 540 (89.3%).

#### Residence

A total of 235 (38.8%) were from residents of halls of individual institutions. On the contrary, 325 (53.7%) respondents were from the category called non-residents of the hall. The least amount of people were in self-accommodated category. 45 (7.4%) participants were in the Self-accommodated category.

## Results

### Evaluation of physiological well-being

Table 2 provides information that among the 605 participants, 319 (52.7%) students became physically sick, and 286 (47.4%) students didn’t become sick when they were newcomers. In the case of the frequency of sickness, the majority of the respondents (N = 347) became sick less than 5 times (57.5%). Unfortunately, 100 (16.6%) participants were sick more than 5 times. On the contrary, 157 students (26%) responded that they had never been sick as newcomers. These data indicated that there an association between being sick in a new environment and being enrolled in universities. Altogether, 332 (54.9%) participants of the total population consulted the physician. However, 273 (45.1%) students reported that they did not consult a doctor for their illness. In the case of medications, 332 (65.1%) students took medicines. However, 211 (34.9%) participants took no medicine (Table 2).

**Table 2:** Demographics of sickness within the university newcomers in Bangladesh

Assessment criteria	Participant count(percentages)
*People who were sick when they were newcomers*	
Yes	319 (52.7%)
No	286 (47.3%)
*Frequency of sickness*	
More than 5 times	100 (16.6%)
Less than 5 times	347 (57.5%)
Never	157 (26%)
*Consultation to physician*	
Yes	332 (54.9%)
No	273 (45.1%)
*Medication*	
Yes	394 (65.1%)
No	211 (34.9%)

**Fig. 1:**
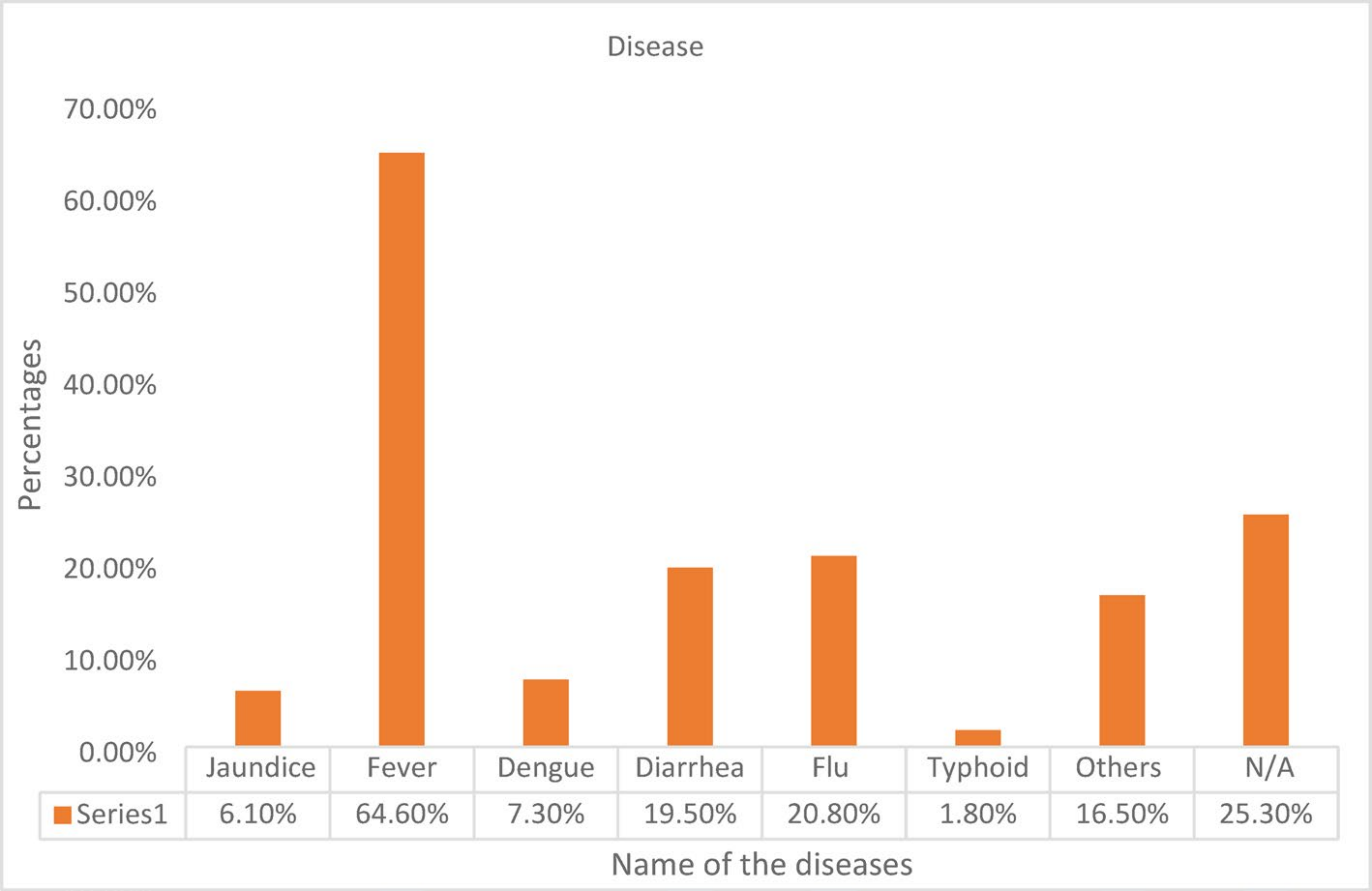
Diseases that affected participants physically (N/A indicated no sickness)

The physical illness of the newcomers was categorized into specific disease cases (Fig. 1) with varying percentages of the diseases that newcomers faced. Fever was the most frequent one found (approximately 65% of cases). Fever is known as the second line of defense and works as a marker for many severe diseases, including typhoid, malaria, COVID-19, pneumonia, ear infection, lung infection, rheumatoid arthritis, and so on. As no further tests were performed by the newcomers, further association with other diseases could not be done. Almost one-quarter of the respondents ensured that they did not become sick as newcomers (Fig. 1). Flu and Diarrhea were most frequent after fever. Flu might be caused by a change in weather, temperature, living place, viral infection, or allergic infection. On the other hand, diarrhea and jaundice indicated poor eating habits and lack of concern about personal hygiene. These two diseases were related to food and water quality. Again, no confirmatory tests were performed to identify whether it was diarrhea or not. So, dysentery, cholera, etc., could also be included here as the symptoms were almost similar for the diseases, and the newcomers could not differentiate, nor did they seek specific tests. A small portion of the participants said that they suffered from diseases other than the given options such as; anemia, thalassemia, diabetes, thyroid problems, tonsilitis, Polycystic ovary syndrome (PCOS), arthritis, psoriasis, piles, spleen operation, appendicitis, kidney operation, scabies, ligament pain, adrenal insufficiency, myasthenia gravis, obesity, blood pressure, dental problem, spinal problem, stomach tumor, vitamin deficiency, allergy or itching, gastric and digestive problems, calcium deficiency, bacterial infections, cold, asthma, allergy, headache, lung problem, migraine, sinusitis, hair problem, viral infection, conjunctivitis, UTI, etc. Most of such cases are likely to be genetic or might be triggered by unfavorable conditions of a new environment.

### Determinants of residential environment quality

After disease cases, emphasis was given to learning the hygienic conditions of the living places of the newcomers. In male participants, 22.5%, 16.1%, and 9.75% of the total population claimed that they are living in places hygienic, hygienic enough, and unhygienic, respectively (Table 3). These percentages were a little bit higher in female participants. 27.9% were found to be living in hygienic conditions, and 18% lived in places that were hygienic enough. Only 5.6% voted that their living place was unhygienic (Table 3). It can be easily anticipated that most females were careful about the hygiene of their living places. This also indicated that the condition of the girls' halls (living accommodation in respective universities) was more hygienic than the boys' halls. Overall, the number of unhygienic living places was very small, and this might have little impact on their physical and mental illness.

**Table 3:** Demographics of living place conditions of the university newcomers in Bangladesh

Sex	Hygienic conditions		
	Hygienic	Hygienic enough	Unhygienic
Male	136 (46.4%)	98 (33.4%)	59 (20.1%)
Female	169 (54.2%)	109 (34.9%)	34 (10.9%)

**Fig. 2:**
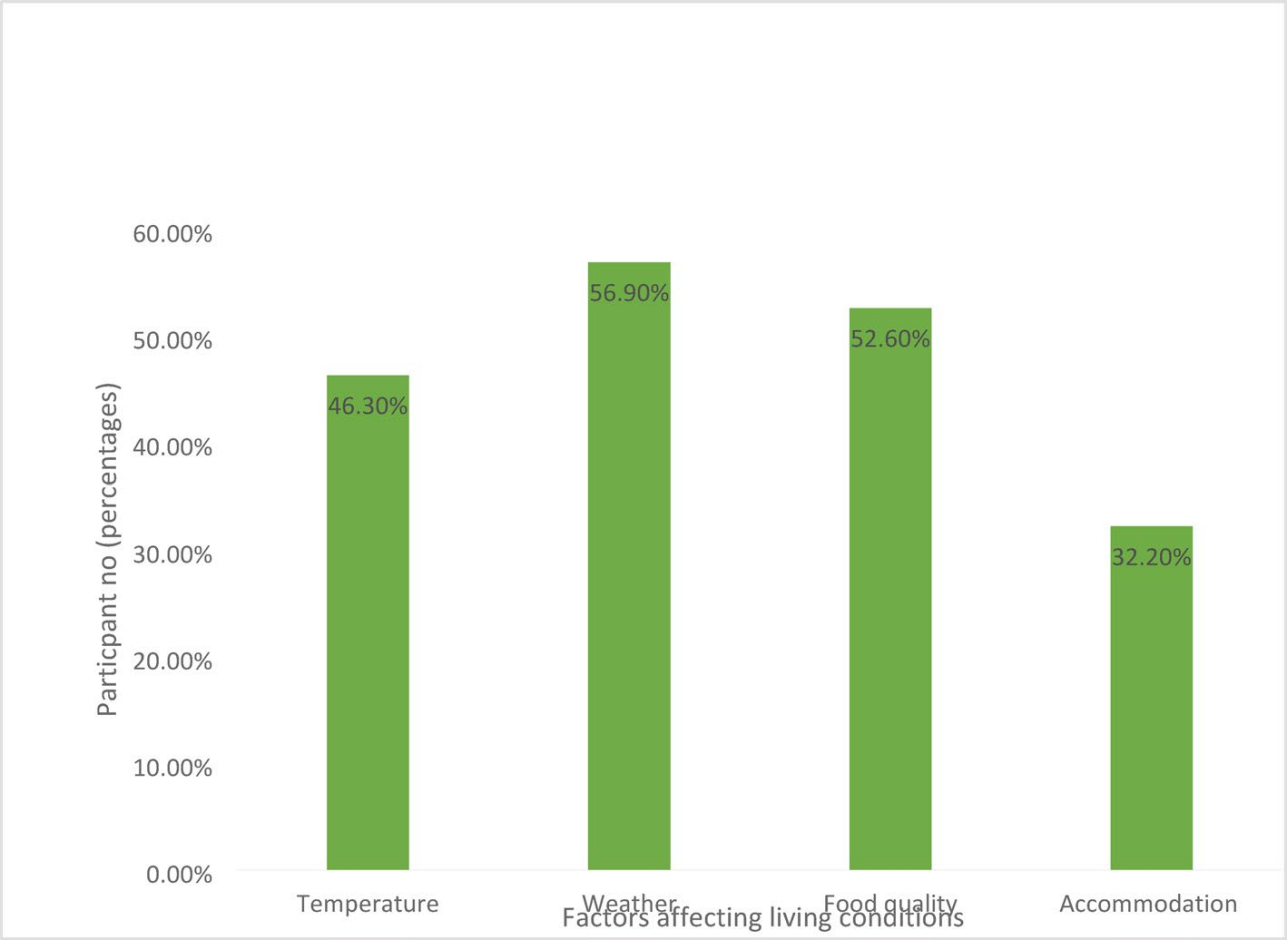
Factors affecting the living conditions of the participants (percentages)

The environmental factors affected everyday living and played a key role in health. Such as temperature, weather, food quality, and accommodation (Fig. 2). The weather was the first reason for being adversely affected, with the majority of respondents in number 343 (56.9%) interviewees. However, some also responded for food quality, temperature, and accommodation labels with approximate percentages of 52.6%, 43.3%, and 31.2%, respectively. All the surrounding factors related to living places had a great impact on each interviewee such as; cold, asthma, allergy, headache, lung problems, migraine, sinusitis, hair problems, viral infection, conjunctivitis, UTI, etc.

### Status of mental well-being

Apart from physical condition, mental health was a matter of concern in our investigation of the newcomers. In number, 493 (81.5%) participants felt pressured as newcomers to universities. A small part of the students did not feel the same (18.5%). Very few (14.2%) consulted with a counselor about their problems, and in opposition, the majority (85.8%) of students never felt the necessity and ignored the fact (Table 4). The percentage of interviewees consulting student advisors of their respective institutes was 31.1%. Again, the majority (68.9%) did not consult at all. Corresponding factors were addressed where only 63.8% of the students admitted to being selected to study at their desired institutes. However, a large percentage (50.4%) of them did not get their desired subjects to pursue their higher studies. Academic pressure related to course load and examinations was another issue. More than half (53.2%) of the respondents could cope with the academic pressure as newcomers (Table 4). Notable changes in circadian rhythm were found in 65.5% of cases, which was more than half of the study population. Changes in circadian rhythm were considered a cause of mental stress by 70% of the respondents, which was opposed by the rest 30% of newcomers. A large group of newcomers (63.8%) denied having enough time for themselves. Half of the respondents (51.7%) were unable to engage in extracurricular activities. Overall, a huge portion (83.3%) were concerned about their mental health status.

**Table 4:** Assessment of the mental condition of the university newcomers in Bangladesh

Assessment criteria	No of attendees(Percentages)
*Do they feel mental pressure?*	
Yes	493(81.5%)
No	112(18.5%)
*Consultation with counselor*	
Yes	86(14.2%)
No	519(85.8%)
*Ever consulted with the student advisor*	
Yes	188(31.1%)
No	417(68.9%)
*Study at the desired institute*	
Yes	386(63.8%)
No	219(36.2%)
*Study at desired subject*	
Yes	300(49.6%)
No	305(50.4%)
*Cope with academic pressure*	
Yes	322(53.2%)
No	283(46.8%)
*Any notable change in circadian rhythm*	
Yes	396(65.5%)
No	209(34.5%)
*Can circadian rhythm change be a cause of mental stress?*	
Yes	423 (70%)
No	182(30%)
*Enough time for self*	
Yes	219(36.2%)
No	386(63.8%)
*Engagement in extracurricular activities*	
Yes	292(48.3%)
No	313(51.7%)
*Concerned about mental health*	
Yes	504 (83.3%)
No	101(16.7%)

**Fig. 3:**
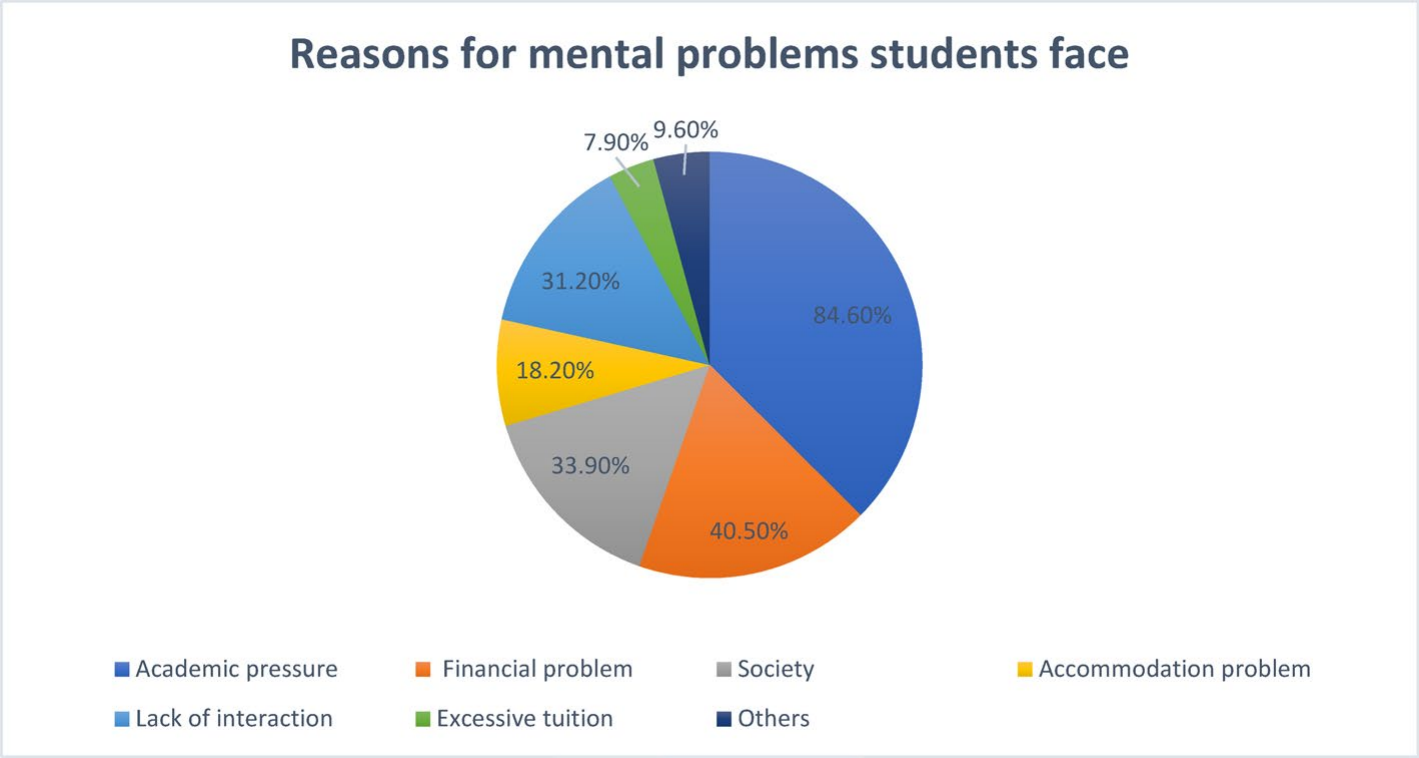
Reasons for mental health problems

Different factors have been addressed for mental health problems (Fig. 3) that the students as newcomers might face and even later in their respective years of university studies. Academic pressure was seen to be the most prominent reason (84.6%). Financial problems (40.5%), society (33.9%), and lack of interaction (31.2%) were also significant factors. Due to the financial crisis, several newcomer students need to conduct tuition of school and college students, and excessive tuition as the cause of mental stress was also on the priority list of some students (7.9%). Some complained about accommodation problems (18.2%) also. Even though the percentages were low, it is undeniable that all these factors were correlated to the severe symptoms of sickness related to mental health in newcomers. Participants themselves mentioned some rare reasons, including overthinking, responsibilities, insecurities, politics, family problems, harassment, relationship issues, criticism, etc., that they mentioned within the “Others” criteria.

**Fig. 4:**
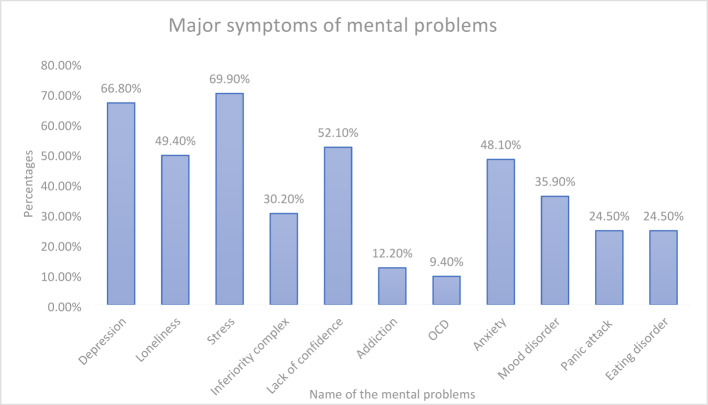
Major symptoms students faced as university newcomers

Adding to our investigation, we tried to know the symptoms of mental problems the newcomer students faced in day-to-day life (Fig. 4) and during the COVID-19 period (Table5). Depression, loneliness, stress, inferiority complex, lack of confidence, addiction, obsessive–compulsive disorder (OCD), anxiety, mood disorder, panic attacks, and eating disorders were the common symptoms the respondents mentioned they struggled with (Fig. 4). Stress (69.9%) and depression (66.8%) were found to be the most prominent ones. In addition, the percentages of lack of confidence (52.1%), loneliness (49.4%), and anxiety (48.1%) could not be ignored. Other symptoms such as mood disorder, panic attacks, inferiority complex, and eating disorders were less than 50%. Because OCD (9.4%) and addiction (12.2%) were quite unfamiliar among people of Bangladesh, the percentages were also quite low among the newcomer students also.

**Table 5:** Information on COVID-19 status within the respondents

COVID-19 Status Respondent count(percentages)	Respondent count(percentages)
*(A)*	
*Affected by COVID-19*	
Yes No	127 (21%) 476 (79%)
*Vaccination*	
Yes No	592 (97.9%) 13 (2.1%)
*Name of vaccine*	
The Pfizer/BioNTech Comirnaty vaccine The Moderna vaccine The Sinopharm vaccine The SII/COVISHILED and AstraZeneca vaccine The Sinovac-CoronaVac vaccine	268 (44.3%) 72 (11.9%) 51 (8.45%) 37 (6.1)
*Doses of vaccine*	
Dose-1 Dose -2 All doses	30 (5%) 298 (49.3%) 277 (45.8%)
*(B)*	
*Did COVID-19 affect mental health*	
Yes	434 (71.7%)
No	171(28.3%)

### Association between gender and selected covariates along with their* p*-values

The data (Table 6) shows statistical significance between males and females in terms of residence, living conditions, mental pressure, academic stress, and lifestyle choices (street food, water intake). More males (57.02%) live in university halls than females (42.98%), while females are more often non-residents (58.46%) than males (41.54%). The distribution is fairly balanced for those who live in self-accommodation, with 53.33% of males and 46.67% of females. A p-value of 0.001 indicates that these differences are statistically significant, meaning there is a meaningful difference between gender and types of residence. In terms of living conditions, more males (63.44%) live in unhygienic places compared to females (36.56%). On the other hand, more females (55.12%) live in hygienic places than males (44.88%). In living conditions that are considered "hygienic enough," both genders show a balanced distribution, with 53.11% of females and 46.89% of males. These differences are also significant between gender and living conditions, with a *p*-value of 0.006. When it comes to mental pressure, more females (56.59%) feel pressured than males (43.41%). Interestingly, 70.54% of males report not feeling mental pressure, compared to 29.46% of females. When asked how often they feel pressure, a larger number of males (73.91%) say they rarely feel any pressure, while more females (53.68%) report feeling pressure all the time. The *p*-values (ranging from 0.000 to 0.046) show that these differences are statistically significant. Regarding academic pressure, 46.73% of females and 53.27% of males say they can manage it. However, more females (57.04%) than males (42.96%) find it hard to cope with academic demands. When asked about mental health, 54.56% of females expressed concerns compared to 45.44% of males. These differences, with p-values of 0.011 and 0.001 respectively, indicate that gender has a significant impact on academic stress and mental health concerns, respectively. Lifestyle choices also show noticeable gender differences. More females (54.30%) eat street food regularly compared to males (45.70%). The *p*-value of 0.006 shows that these lifestyle differences exist. Meanwhile, more males (66.39%) drink tap water, while fewer females (33.61%) do so. The p-value of 0.001 shows that these differences are statistically significant.

**Table 6:** Association between gender and selected covariates along with their *p*-values

Variables	Gender		Chi-Square Value	*p*-value
	Female 312/605 (51.6%)	Male 293/605 (48.4%)		
*Residence*				
Resident of Hall Non-Resident of Hall Self-Accommodated	101/235 (42.98%) 190/325 (58.46%) 21/45 (46.67%)	134/235 (57.02%) 135/325 (41.54%) 24/45 (53.33%)	13.56	0.001
*People who were sick when they were newcomers*				
Yes No	163/319 (51.10%) 149/286 (52.10%)	156/319 (48.90%) 137/286 (47.90%)	0.06	0.805
*Demographics of living place conditions of the university newcomers*				
Unhygienic Hygienic Hygienic enough	34/93 (36.56%) 167/303 (55.12%) 111/209 (53.11%)	59/93 (63.44%) 136/303 (44.88%) 98/209 (46.89%)	10.11	0.006
*Do they feel mental pressure?*				
Yes No	279/493 (56.59%) 33/112 (29.46%)	214/493 (43.41%) 79/112 (70.54%)	26.89	0.000
*How often do you feel pressured?*				
Never Always Rarely Often	6/23 (26.09%) 51/95 (53.68%) 22/48 (45.83%) 233/439 (53.08%)	17/23 (73.91%) 44/95 (46.32%) 26/48 (54.17%) 206/439 (46.92%)	7.18	0.046
*Cope with academic pressure*				
Yes No	150/321 (46.73%) 162/284 (57.04%)	171/321 (53.27%) 122/284 (42.96%)	6.42	0.011
*Concerned about mental health*				
Yes No	275/504 (54.56%) 37/101 (36.63%)	229/504 (45.44%) 64/101 (63.37%)	10.83	0.001
*Did COVID-19 affect mental health?*				
Yes No	240/434 (55.30%) 72/171 (42.11%)	194/434 (44.70%) 99/171 (57.89%)	8.55	0.003
*Street food*				
Yes No	265/488 (54.30%) 47/117 (40.17%)	223/488 (45.70%) 70/117 (59.83%)	7.55	0.006
*Water you intake*				
Tap water Potable water	41/122 (33.61%) 271/483 (56.11%)	81/122 (66.39%) 212/483 (43.89%)	19.74	0.001

## Medical facilities at universities for physical and mental care of newcomers

Despite it being visible that the majority of the respondents were sick both physically and mentally during their initial years of universities, the available facilities at medical centers of respective institutions were not adequate. It was mentioned by the interviewees that this sector of almost all possible institutions was not at all suitable for the students' overall health. Thus, a major part of the participants (46.4%) ignored visiting medical centers and thought about other options outside the universities. Only 53.5% of the participants visited the medical centers, and the majority (approximately 33%) did not receive proper treatment. Those who tried to reach them mentioned various drawbacks of those facilities such as not taking the proper time to listen to the concerns of the students, not monitoring the actual cause of the sickness, unequipped testing labs, malfunctioning of diagnostic sophisticated equipment, absence of psychologists/psychotherapists, lack of a friendly environment for the sick students and even rough and rude behavior towards students who seek their attention.

## Effect of Covid-19

The interviewees were also questioned to gather information about their physical and mental condition during COVID-19 (Table 5). The number of affected participants was 127 (21%), while a large portion (79%) denied the fact. In total, 592 (97.9%) participants got vaccinated. Vaccinated participants took a wide range of vaccines of different genera. The Pfizer / BioNTech vaccines were common and received by 44.3% of respondents. The Sinopharm vaccine, the Moderna vaccine, the SII/COVISHILED and AstraZeneca vaccine, and the Sinovac-CoronaVac vaccine represented 29.3%, 11.9%, 8.45%, and 6.1%, respectively. The majority (95.1%) of respondents were vaccinated with the second and booster dose. Only 4.9% of the vaccinated participants have taken the first dose.

According to the statistics on the mental health of the participants during COVID-19, 434 (71.7%) participants ensured their mental health had deteriorated drastically. Left of the respondents did not feel the same.

## Discussion

Our investigation was one of the earliest kinds of endeavors to learn the actual physical and mental condition of university newcomers and to associate their illness with varying factors. Different reasons were identified for being ill physically, such as living conditions, personal hygiene practices, food habits, etc. All of the participants gave different opinions on their mental health condition. One of the concerning reasons was not being able to adjust easily to the academic pressure of university.

More than half of the participants experienced fever (64.60%) as newcomers. This can be associated with other diseases or might be because of not being able to adjust to the new environment. No previous study showed a correlation of this symptom with other physical or mental conditions only dedicated to university newcomers in Bangladesh. There were reports where specifically fever associated with dengue has been addressed among university students [19]. In another investigation, the overall health condition of university students has been addressed specifically correlated to students' health, living place, smoking habits, food habits, and vaccination of various diseases like BCG, DPT, etc. [20]. In some cases, physical health conditions were correlated to anxiety and depression among Bangladeshi university students [14]. However, no conclusive data on physical and mental health and associated factors with strategic solution possibilities have been obtained.

In our study, there were findings of food and water-borne diseases like diarrhea and jaundice. This indicates that either the quality of food and water they intake is not so good or they are not concerned about maintaining personal hygiene. A very small number of students responded that they had a particular previous disease history like anemia, thalassemia, diabetes, tonsillitis, thyroid problem, arthritis, PCOS, psoriasis, piles, spleen operation, appendicitis, scabies, kidney operation, ligament pain, adrenal insufficiency, myasthenia gravis, obesity, blood pressure, dental problem, spinal problem, stomach tumor, etc. However, these previous diseases did not play any major role in the case of being sick as newcomers. Another analysis denoted that those socio-economic demographics (e.g., schooling, residence, employment status) and various chronic health conditions such as hypertension, hypercholesterolemia, diabetes mellitus, and osteoarthritis severely affect positive mental health [21]. They covered the age group starting from 45 years old with no upper boundary of the range and was conducted in Spain. Previous to this investigation, no other report has been published for university students or newcomers in Bangladesh specially on their health status wellbeing and therefore, it justifies the urgent need for such a study in Bangladesh. This transition period has a greater impact on the later life of a student – either positively or negatively depending upon the circumstances. This survey might serve as a good reference for any type of study regarding university students' physical health and diseases as there is no previous case study found that relates to this till now.

Physical and Mental health problems are highly interconnected. "No health without mental health" is what is said when it comes to the concept of correlating these two. [22] Approximately 71.7% of the respondents felt various symptoms of mental sickness. Almost all the participants (81.5%) felt mentally pressured most of the time. This statistic can be compared with the Bangladeshi university students’ sample[23]. However, the percentage was seen far higher in our study. Academic pressure is a burden for a large number of newcomers. Financial problems, societal pressure, accommodation problems, lack of interaction, and excessive tuition also had greater impacts on feeling mentally pressured. Some rare reasons retrieved were overthinking, responsibilities, insecurities, politics, family problems, harassment, relationship issues, criticism, etc. Studies such as the causes of mental health problems also mentioned such criteria [24, 25]. Even though a large number of students had moderate to very friendly relationships with their parents, they claimed not to be able to interact with people easily. Studies considering the pattern of friends, family, and community also claimed a similar pattern [26]. Respondents did not feel the urge to consult the counselor and student advisor for mental and academic issues, respectively. It is undeniable that a student counselor and advisor plays a vital role in the mental well-being and developmental activities of a student. From the study, it can be anticipated that they do not even care about mental health problems because of a lack of awareness about mental well-being. Awareness of mental health is as important as physical health [27]. Ignoring the fact that mental health is related to physical health leads to severe problems [28]. Although the majority of the participants were studying at reputable institutions in Bangladesh, they were not satisfied with their choice of subjects or departments. Depression, stress, lack of confidence, inferiority complex, loneliness, anxiety, mood disorder, and eating disorder were found as the major causes of mental problems. There, it can be seen that these symptoms often lead to various difficulties in day-to-day life[29, 30]. Because OCD and anxiety are quite unfamiliar among people of Bangladesh, the percentage was quite low. Medicine for mental sickness seems to be quite ignorable among the respondents. It is suggested to take anti-depressants, anti-anxiety, antipsychotics, mood stabilizing, and stimulants when necessary [28]A lot of participants hesitated or simply could not share problems with others. They claimed to control their emotion, and as was seen previously, they preferred not to share problems with anyone; they seemed to keep all problems to themselves. Although the largest portion agreed that social media affects daily life, a large portion was still confused about this. The result was comparable to other studies[31]. A notable change was seen in the circadian rhythm, but most of the participants did not think or were confused that it can be a cause of mental stress. A greater portion of participants claimed they did not have enough time for what they love to do and for extracurricular activities. Extracurricular activities play a very important role in academic achievement, character, social development, community involvement, and all [32]. The majority claimed to be concerned about their mental situation. However, this was not reflected in previous inquiries, which can be erroneous. What is more concerning is that, a two to threefold increased death rate can be observed in people with chronic mental sickness. Abnormal obesity, metabolic syndrome, COPD (Chronic Obstructive Pulmonary Disease), cancer, diabetes, dental diseases, migraine, insomnia, arthritis, chronic back/ neck pain, stroke, asthma, ulcer, vulnerability towards HIV, Hepatitis, Tuberculosis etc., are seen to be commonly found in patients with mental health disorders. In a study, it was observed that, in USA individuals with severe anxiety, depression, extreme stress, OCD, mood disorder are more likely to get acquainted with elevated odds of physical pain like; gastrointestinal disease, vision and hearing problems, frequent sick days, severe allergic reactions, worse physical functioning, and poor oral health etc. This clearly interprets mental health disorders, and their proper treatment is more than how it is neglected, and consciousness is a must. [22]

The majority of respondents (79%) were never affected by COVID-19, as the information was taken after the pandemic and mass vaccination period. However, this result may be erroneous as information about confirmatory tests was not collected. The information was taken from November 2022 to April 2023, and the statistic shows a static curve. Almost ignorable numbers of new cases arose at that time. Almost all of the students (97.9%) were vaccinated. This is a very positive result for such a developing country as Bangladesh. A timely programmed and maintained vaccination system helped the country to deal with this greatly. Our study showed the Pfizer/ BioNTech Comirnaty vaccine was taken by the majority of the participants (44.3%). More than half of the interviewees thought that their mental health was affected by the COVID-19 pandemic. The pandemic severely deteriorated the mental condition of the students, and it is similar to our study.

## Suggestions for upcoming newcomers

Important suggestions given by the participants for upcoming newcomers are: Participating in extracurricular activities; being strong and positive in every situation; counselling at regular basis; trying to cope with new situations; listening to music, learning new things; meeting new friends; regular contact and sharing of everything with parents; in the case of hesitation, sharing with close or best friend(s); giving importance to mental health; finding out proper time for self, not being anxious in any bad situation, and taking time to adjust to everything; improving the relationship between teacher and students; exploring and visiting new places; reading books, and connecting with different clubs in university; eating safe foods and drinking plenty of water; not to hesitate for asking help; exercising, properly sleeping, relaxation, and recreation; compromise and always stick to own morals even though opinions do not match; taking a little break from everything, refreshing mind and again concentrating on the right track; not to be involved in politics; freshers need friendly academic counselor(s); improvement of hall facilities; ensuring hygienic accommodations for hall residents; not taking too much stress after giving the hundred percent of the ability; keeping faith in the Almighty; working with own hands as half of the brain's cortex is dedicated to the hands and so hands-on hobby gives the brain a good workout; creating a schedule for every work; keep physical health good, giving importance to mental health as these are interconnected; improvement of medical centers. These suggestions from the respondents opened up different ways for the newcomers to combat the situation and also provided a priority list for the universities in Bangladesh.

## Limitations

This study had some limitations. Firstly, the questionnaire was provided to the participants in person as well as via social media. Many of the social media users did not fill up the form or filled it up incompletely. Besides, at the beginning of the study, some of the respondents did not understand a few questions. So, those questions were modified a little for their convenience. The questionnaire was provided in person only within the University of Dhaka. A homogenous distribution of data collection within the age ranges and sessions was missing. In the mental health condition part, self-assessments were taken, which might not be completely accurate for all the cases. Most of the participants responded that they were affected by COVID-19, but as there was no question of whether they tested it or not, this information might not be confirmatory. This survey thus may serve as a useful measure for further studies on this timely issue, but it might not be taken out of context and should not be compared with how students feel now as this study covers a very short period.

## Conclusion

This study was the first endeavor to examine the overall physical and mental health condition of university newcomers in different universities in Bangladesh. The study showed clearly that university freshmen in Bangladesh experienced a sudden and huge change in their overall health condition. This survey portrayed the illnesses, specific reasons, symptoms, and how to overcome the problems for both physical and mental health conditions. Factors affecting physical and mental health, the condition of the places they live, the safety of the foods and water they intake, and the necessity of raising awareness about mental health were also portrayed. A crystal-clear view of each type of disease, medicine, type and methods of medications, emotional control, and interaction with others could not be assessed in this study. This broad-spectrum study covered every possible reason and case of illness of respondents. The COVID-19 pandemic and its aftereffects on both physical and mental health conditions were recognized through this survey, which showed a very positive aspect for a developing country like Bangladesh. Suggestions of the participants showed many important measures and topics which will be of great impact for the upcoming newcomers. As no previous publication or study directly related to this issue was found, it can be said that this study is going to be a baseline and milestone in any type of further studies related to the health condition of university newcomers. If the findings, possible reasons, results, and suggestions are gone through and applied properly, then they will have a great and positive impact on the lifestyle, educational system, and lastly, on the development of the whole country on a huge scale.

## Supplementary Information

Below is the link to the electronic supplementary material.

Supplementary Material 1

Supplementary Material 2

## Data Availability

Data is provided within the manuscript and within supplementary information files.
