# Supplementary material for: A cross-sectional study on the physical and mental health condition of university newcomers in Bangladesh
Source: Discov Ment Health. 2025 Aug 20;5(1):129. doi: 10.1007/s44192-025-00276-5 (PMC12367575; doi:10.1007/s44192-025-00276-5)
Supplement: Supplementary file 1 — Supplementary Material 1 [file 44192_2025_276_MOESM1_ESM.pdf]

ডিন অফিস
জীববিজ্ঞান অনুষদ
ঢাকা বিশ্ববিদ্যালয়, ঢাকা-১০০০, বাংলাদেশ

Tel : 58613243
PABX : 9661900-59/4355, 7493
Fax : 880-2-9667222
E-mail : deanbio@du.ac.bd
akhterkhan@du.ac.bd

Ref. No. 275/Biol. Scs.

September 25, 2024

Ethical Review Committee

Professor Dr. Munawar Sultana
Department of Microbiology
Dhaka University, munawar@du.ac.bd

Sub: Ethical Clearance

Dear Dr. Munawar Sultana,

With reference to your application on the above subject, this is to inform you that your research proposal entitled "A Comparative Study on the Physical and Mental Health Condition of University Newcomers in Bangladesh" has been reviewed and approved by the Ethical Review Committee of the Faculty of Biological Sciences, University of Dhaka.

I wish for the success of your research project.

akhter 25/9/24

Professor Dr. Md. Akhter Hossain Khan
Dean (Acting), Faculty of Biological Sciences
University of Dhaka

CS CamScanner
